# Mercury Exposure in Munduruku Indigenous Communities from Brazilian Amazon: Methodological Background and an Overview of the Principal Results

**DOI:** 10.3390/ijerph18179222

**Published:** 2021-09-01

**Authors:** Paulo Cesar Basta, Paulo Victor de Sousa Viana, Ana Claudia Santiago de Vasconcellos, André Reynaldo Santos Périssé, Cristina Barroso Hofer, Natalia Santana Paiva, Joseph William Kempton, Daniel Ciampi de Andrade, Rogério Adas Ayres de Oliveira, Rafaela Waddington Achatz, Jamila Alessandra Perini, Heloísa do Nascimento de Moura Meneses, Gustavo Hallwass, Marcelo de Oliveira Lima, Iracina Maura de Jesus, Cleidiane Carvalho Ribeiro dos Santos, Sandra de Souza Hacon

**Affiliations:** 1 Departamento de Endemias Samuel Pessoa, Escola Nacional de Saúde Pública, Fundação Oswaldo Cruz (ENSP/Fiocruz), Rua Leopoldo Bulhões, 1480-Manguinhos, Rio de Janeiro 21041-210, Brazil; aperisse41@gmail.com (A.R.S.P.); sandrahacon@gmail.com (S.d.S.H.); 2 Centro de Referência Professor Hélio Fraga, Escola Nacional de Saúde Pública, Fundação Oswaldo Cruz (CRPHF/ENSP/Fiocruz), Estrada de Curicica, 2000-Curicica, Rio de Janeiro 22780-195, Brazil; paulovictorsviana@gmail.com; 3 Laboratório de Educação Profissional em Vigilância em Saúde, Escola Politécnica de Saúde Joaquim Venâncio, Fundação Oswaldo Cruz (EPSJV/Fiocruz), Av. Brazil, 4365-Manguinhos, Rio de Janeiro 21040-900, Brazil; anacsvasconcellos@gmail.com; 4 Instituto de Pediatria e Puericultura Martagão Gesteira, Faculdade de Medicina, Universidade Federal do Rio de Janeiro (UFRJ), Rua Bruno Lobo, 50-Cidade Universitária, Rio de Janeiro 21941-912, Brazil; cbhofer@hucff.ufrj.br; 5 Instituto de Estudos em Saúde Coletiva (IESC), Universidade Federal do Rio de Janeiro (UFRJ), Avenida Horácio Macedo, s/n, Ilha do Fundão-Cidade Universitária, Rio de Janeiro 21941-598, Brazil; natalia_uff@hotmail.com; 6 Faculty of Medicine, Imperial College London, Medical School Building, St Mary’s Hospital, Norfolk Place, London W2 1PG, UK; kemptonj@hotmail.com; 7 Centro de Dor, Departamento de Neurologia, Hospital das Clínicas, Faculdade de Medicina, Universidade de São Paulo (USP), Av. Dr. Enéas Carvalho de Aguiar, 255-Cerqueira César, São Paulo 05403-000, Brazil; ciampi@usp.br (D.C.d.A.); roger.adas.doc@gmail.com (R.A.A.d.O.); 8 Programa de Pós-Graduação em Psicologia Clínica do Instituto de Psicologia da Universidade de São Paulo (USP), Av. Professor Mello Moraes, 1721-Butantã, São Paulo 05508-030, Brazil; rafa.achatz@gmail.com; 9 Laboratório de Pesquisa de Ciências Farmacêuticas (LAPESF), Centro Universitário Estadual da Zona Oeste (UEZO), Av. Manuel Caldeira de Alvarenga, 1.203, Rio de Janeiro 23070-200, Brazil; jamilaperini@yahoo.com.br; 10 Programa de Pós-Graduação em Ciências da Saúde (PPGCSA), Universidade Federal do Oeste do Pará, Rua Vera Paz Av. Vera Paz, s/n, Bairro Salé, 1° Pavimento, Bloco Modular Tapajós, Unidade Tapajós, Santarém, Pará 68035-110, Brazil; heloisa.meneses@ufopa.edu.br; 11 Programa de Pós-Graduação em Biociências (PPGBio), Universidade Federal do Oeste do Pará, Rua Vera Paz, s/n, Bairro Salé, Santarém 68035-110, Brazil; gustavo.hallwass@gmail.com; 12 Seção de Meio Ambiente, Instituto Evandro Chagas, Secretaria de Vigilância em Saúde, Ministério da Saúde (SEAMB/IEC/SVS/MS), Rodovia BR-316 km 7, s/n, Levilândia 67030-000, Brazil; marcelolima@iec.gov.br (M.d.O.L.); iracinajesus@iec.gov.br (I.M.d.J.); 13 Distrito Sanitário Especial Indígena Rio Tapajós (DSEI), Secretaria Especial de Saúde Indígena Tapajós (Sesai), Av. Santa Catarina, 10° Rua, nº 96, Bairro Bela Vista, Itaituba 68180-210, Brazil; cleidiane.santos@saude.gov.br

**Keywords:** environmental pollution, mercury exposure, indigenous people, Brazilian Amazon, gold mining, fish, children health, neurological effects, genetic polymorphism, *ALAD*, illegal mining activities

## Abstract

The Amazonian indigenous peoples depend on natural resources to live, but human activities’ growing impacts threaten their health and livelihoods. Our objectives were to present the principal results of an integrated and multidisciplinary analysis of the health parameters and assess the mercury (Hg) exposure levels in indigenous populations in the Brazilian Amazon. We carried out a cross-sectional study based on a census of three Munduruku indigenous villages (*Sawré Muybu, Poxo Muybu,* and *Sawré Aboy*), located in the *Sawré Muybu* Indigenous Land, between 29 October and 9 November 2019. The investigation included: (i) sociodemographic characterization of the participants; (ii) health assessment; (iii) genetic polymorphism analysis; (iv) hair mercury determination; and (v) fish mercury determination. We used the logistic regression model with conditional Prevalence Ratio (PR), with the respective 95% confidence intervals (CI95%) to explore factors associated with mercury exposure levels ≥6.0 µg/g. A total of 200 participants were interviewed. Mercury levels (197 hair samples) ranged from 1.4 to 23.9 μg/g, with significant differences between the villages (Kruskal–Wallis test: 19.9; *p*-value < 0.001). On average, the general prevalence of Hg exposure ≥ 6.0 µg/g was 57.9%. For participants ≥12 years old, the Hg exposure ≥6.0 µg/g showed associated with no regular income (PR: 1.3; CI95%: 1.0–1.8), high blood pressure (PR: 1.6; CI95%: 1.3–2.1) and was more prominent in *Sawré Aboy* village (PR: 1.8; CI95%: 1.3–2.3). For women of childbearing age, the Hg exposure ≥6.0 µg/g was associated with high blood pressure (PR: 1.9; CI95%: 1.2–2.3), with pregnancy (PR: 1.5; CI95%: 1.0–2.1) and was more prominent among residents in *Poxo Muybu* (PR: 1.9; CI95%: 1.0–3.4) and *Sawré Aboy* (PR: 2.5; CI95%: 1.4–4.4) villages. Our findings suggest that chronic mercury exposure causes harmful effects to the studied indigenous communities, especially considering vulnerable groups of the population, such as women of childbearing age. Lastly, we propose to stop the illegal mining in these areas and develop a risk management plan that aims to ensure the health, livelihoods, and human rights of the indigenous people from Amazon Basin.

## 1. Introduction

Mercury (Hg) is a heavy metal ubiquitously distributed in the environment [1]. Although humans have used this metal since ancient times [2], the increasing use of mercury in the last decades has caused important changes in their biogeochemical cycle and, consequently, the risk of becoming ill due to exposure to this contaminant has become a public health concern [3,4,5,6].

In Brazil, studies developed since the 1980s point out that the artisanal small-scale gold mining activities (ASGM) in the Amazon are the main cause of environmental contamination by mercury in the region [7,8,9,10,11,12]. Over the last 50 years, the ASGM have released thousands of tons of mercury into the Amazonian environment and, simultaneously, contributed to the process of soil and sediment erosion that facilitates the mobilization of natural mercury [2,13,14]. In a recent study conducted by Teixeira et al. [15], mineralogical analysis carried out on soil samples from the region of *Cachoeira do Piriá* (state of Pará) revealed high concentrations of mercury, above the limit established by Brazilian environmental legislation, despite the absence of mineral formations constituted by mercury (i.e., despite the absence of natural mercury). These results make even more evidently the contribution of the ASGM (also called *garimpo*) to environmental mercury contamination and indicate that the presence of natural mercury in the Amazon’s soil is not homogenous.

Moreover, the impact of gold mining on human mercury exposure has been evaluated in several cross-sectional studies involving control groups (i.e., populations living in areas with no history of mining activity) [16,17,18,19,20]. These studies reveal that the mercury levels detected in hair samples collected from groups living in areas impacted by mining are considerably higher than the levels observed in control groups. Such scientific findings strengthen the hypothesis that the primary source of human mercury contamination in the Brazilian Amazon is from mining activities, despite the natural mercury in the soil.

According to a map published by RAISG (Georeferenced Social and Environmental Information Network on Amazon), there are 453 illegal mining sites in the Brazilian Amazon. In the entire Amazonian ecosystem—present in nine Latin American countries—there are more than 2500 illegal mining sites spread over a territory of 7 million km^2^ [21]. This scenario of environmental destruction has had severe ramifications for the health and lives of the traditional populations living in the Amazon, especially on indigenous peoples, which are considered one of the keenest consumers of fish in the world [22,23,24]. As a result of the high fish consumption, these populations are at a greater risk of mercury intake above the safe limits established by the Food and Agriculture Organization/World Health Organization (FAO/WHO) [25] (i.e., 1.6 µg/kg bw/week for children, woman of childbearing age and pregnant woman, and 3.2 µg/kg bw/week for adults in general) and by the United States Environmental Protection Agency (U.S.EPA) [26] (i.e., 0.1 µg/kg bw/day). The Hg released by the ASGM undergoes methylation in the aquatic environment, mediated by microorganisms, transforming it into methylmercury (MeHg) [27]. MeHg is the most dangerous form of mercury to human health due to its deleterious effects on the central nervous system and its biomagnification up aquatic trophic chains [28,29,30].

The principal human exposure route to MeHg is the consumption of contaminated aquatic organisms, such as fish, shrimp, and crabs. To support this statement, numerous studies [31,32,33,34,35,36,37,38,39,40,41,42,43,44] carried out in the Amazon basin have revealed mercury concentrations in different fish species above the limits to commercialization established by FAO/WHO [45] (i.e., 0.5 µg MeHg/g for non-piscivorous fish and 1.0 µg MeHg/g for piscivorous fish). As a direct consequence of this fact, many scientific studies have been shown hair mercury concentrations from traditional Amazonian people that correspond to methylmercury intake in amounts several times greater than the safe limits recommended by FAO/WHO [25] and U.S.EPA [26], as we can see in some review papers [5,46,47].

In addition, the construction of dams for the operation of hydroelectric plants significantly affects the mercury cycle in the Amazon environment, as it facilitates the mercurial methylation process and other chemical transformations (e.g., demethylation, reduction, and oxidation) [48,49,50]. In general, dams have favorable physicochemical conditions (e.g., temperature, oxygenation, redox potential, dissolved organic matter) for the proliferation of bacteria that participate in mercury cycling, promoting an increase in the concentration of dissolved organic matter which serves as a carbon source for the organomercurial species, and additionally, inhibits fish migration, facilitating the bioaccumulation and biomagnification of the methylmercury produced [50,51,52,53].

Another significant challenge is mercury exposure during the prenatal period. Women’s MeHg exposure during pregnancy can cause severe damage to the fetus’s central nervous system, and some of these damages can be irreversible. Studies have associated prenatal MeHg exposure with cognitive delays and mild mental retardation [6,54,55]. Reuben et al. [56] observed loss of cognitive ability in ASGM mercury-exposed children in the Peruvian Amazon. In Brazil, Marques et al. [57,58] identified psychomotor alterations and mental development problems in children from Rondônia state, in the Amazon region.

Recent studies regarding the effect of mercury on adult’s health revealed emotional changes, such as depression and aggressiveness, motor problems [59], and changes in the visual field [60]. Furthermore, some studies have demonstrated an association between MeHg exposure with the development of neurodegenerative diseases such as Alzheimer’s and Parkinson’s disease [61,62].

Considering the Tapajós River basin, an area inhabited by the Munduruku people and other indigenous groups in the Brazilian Amazon, some studies have analyzed mercury levels in environmental samples [5,13,35,41,63,64,65]. Almost all of the revised studies detected mercury in relevant concentrations in different environmental samples such as water, sediment, plankton, and macrophytes fish [5,13,35,41,63,64,65]. Nevertheless, only three studies are available on the scientific indexed bases regarding mercury exposure in the Munduruku indigenous territory [66,67,68].

The Munduruku indigenous that live in the *Sai Cinza* Indigenous Land, for example, have shown high mercury levels in hair samples (14.45 μg/g for children from 7 to 12 years old, 15.70 μg/g for women between 14 and 44 years old, and 14.1 μg/g for the remaining population) [67]. The average levels detected were higher than the safe limit (6 μg/g) established by WHO [69]. During an analysis of 365 samples of fish from the Tapajós basin, Dórea et al. [68] revealed that, in general, 26% of fish Hg concentrations were above 0.5 µg/g, and 11% were above 1.0 µg/g, surpassing the maximum reference limits to commercialization of the fishes by FAO/WHO [45]. Notwithstanding, the biomarker of fish consumption (Hair-Hg) was significantly higher in the Kayabi indigenous group (12.8 µg/g) that shares part of the territory with the Munduruku, who showed a mean mercury hair concentration of 3.4 µg/g. In turn, Brabo et al. [66] also pointed out that the average concentration of mercury in the carnivorous species of fish consumed in the upper Tapajós region was 0.293 μg/g.

The abovementioned studies demonstrate that mercury exposure is a long-term and persistent problem in the region, representing a severe risk for the population. Despite the undeniable relevance of this topic in the public health agenda, there is a noticeable scarcity of information about mercury exposure in the Munduruku group as well as in other indigenous people living in the Amazon. In the last two years, the Munduruku have suffered as a consequence of the anti-environmental policies proposed by the Brazilian government, especially Bill 191/2020, that aims to regulate the mining activities in protected areas of Amazon, including Indigenous Lands and Conservation Units [70]. Beyond the Munduruku, the Yanomami are also under violent attack with the invasion of the traditional territories and rights violation, but also with threats, physical attacks, setting fire to Indigenous leaders’ homes, shootings, and deaths [71].

With this critical scenario in mind, the primary objective of this article is to present the methodological background, as well as to share an overview of the principal results, including the sociodemographic characterization, the general health situation in the villages, and to assess the chronic mercury exposure in the Munduruku Indigenous people, living in the Middle-Tapajos Region, Brazilian Amazon.

## 2. Materials and Methods

### 2.1. Research’s Background

**Figure 1:**
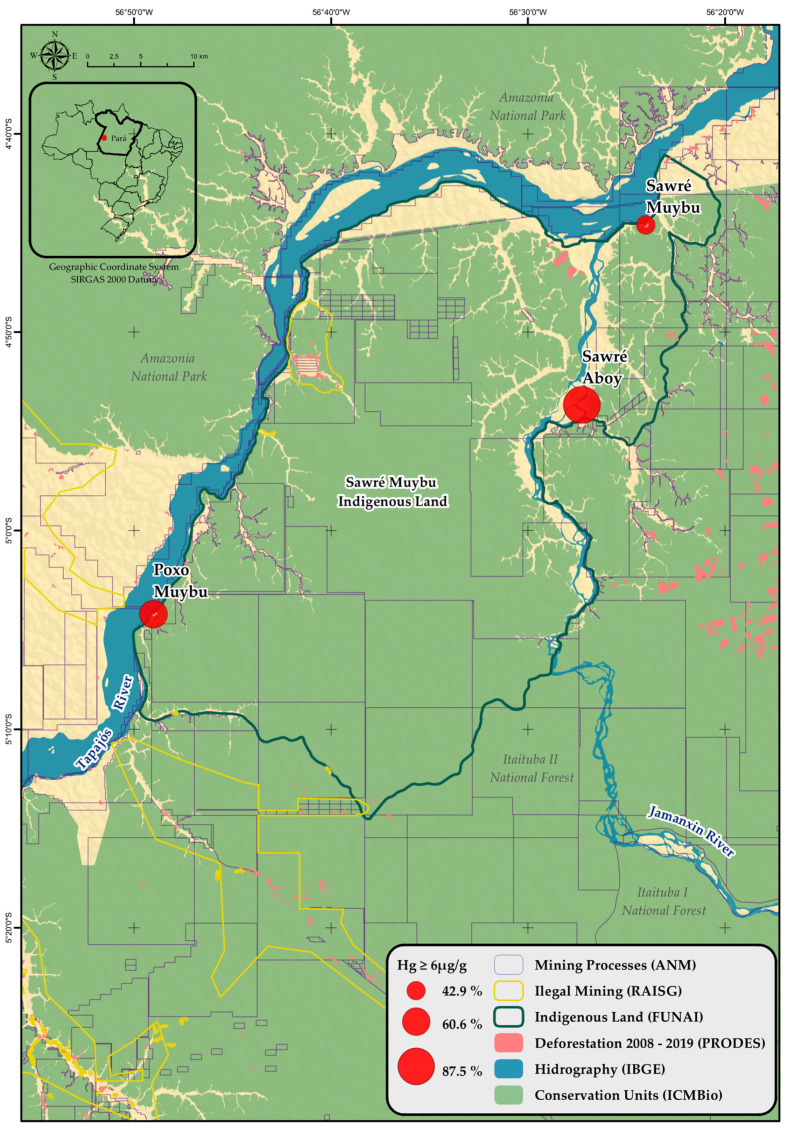
Study area highlighting the investigated villages: *Sawré Muybu* (downstream), and *Poxo Muybu* (upstream) on the margins of Tapajós River, and *Sawré Aboy* (downstream) on the margins of Jamanxin River. Polygons with gray lines correspond to areas with legal requirements for mining. Polygons with yellow lines correspond to illegal mining activities. *Sawré Muybu* Indigenous Land, Pará, Amazon, Brazil, 2019.

Given mercury’s ability to contaminate the environment, as described in the previous section, and the increasing presence of *garimpeiros* (gold miners) in the area (as can be seen in Figure 1), the *Pariri* Indigenous Association (representing the Munduruku indigenous people living in the Middle-Tapajós Region) sent a letter to the Oswaldo Cruz Foundation to request an assessment of mercury contamination in their Indigenous territory. In response, a multidisciplinary, specialized team was formed to put together a comprehensive plan of action and then to deliver the fieldwork.

### 2.2. Population and Study Area

The Munduruku people are linked to the Tupi linguistic branch, in the Munduruku linguistic family. As people of a warrior tradition, the Munduruku have culturally dominated the Tapajós valley region, which in the early days of contact and during the 19th century was known as *Mundurukânia*. Today, the group inhabits lands located in the states of Pará, Amazonas, and Mato Grosso. These people are concentrated mainly in savanna regions of the Amazon rainforest, on the banks of navigable rivers [72,73].

The *Sawré Muybu* Indigenous Land (IL) (also known as *Pimental*), which the Munduruku people traditionally occupy, spans only 178,173 hectares and is located in the municipalities of *Itaituba* and *Trairão*, in the state of Pará (Figure 1). The area’s demarcation process began in 2007, but the demarcation process remains unfinished. In the area covered by the *Sawré Muybu* IL, there are eight indigenous villages summing up almost 800 people.

### 2.3. Study Design

A cross-sectional study was carried out in three villages selected from *Sawré Muybu* IL (*Sawré Muybu, Poxo Muybu*, and *Sawré Aboy*), according to the request of the *Pariri* Indigenous Association (Figure 1). In the designated villages, we conducted a population census, and all residents were invited to participate in the study. There was no refusal and, therefore, no probabilistic sampling methods were used to include participants.

### 2.4. Fieldwork Steps

The fieldwork investigation was carried out by a multidisciplinary and specialized team between 29 October and 9 November 2019, during the dry season. The investigation included: (i) sociodemographic characterization of the participants (i.e., family income, schooling, family composition, physical structure of the houses); (ii) health assessment (i.e., anthropometric indicators, estimates of the prevalence of chronic non-communicable diseases, and sexually transmitted diseases, as well as neurological and child development evaluations); (iii) genetic polymorphism analysis; (iv) hair mercury determination; and (v) fish mercury determination.

### 2.5. Sociodemographic Characterization

We organized the individual survey questionnaire in four sections:

(a) The first section (head of household’s data) characterizes the physical and demographic structure of the homes visited, including the length of residence, materials used for building the house, sources of drinking water and presence of bathroom, occupational activities, income, social benefits, number of years in education for the head of household, and the dietary pattern of inhabitants, with an emphasis on fish consumption.
(b) The second section (family members’ data) characterizes the health of the family and the community, including a history of previous hospitalizations; previous Malaria treatment, and history of contact with mercury from *garimpos*.
(c) The third section (women’s health data) questionnaire addressed reproductive history, the number of children, pregnant, and breastfeeding status.
(d) The fourth section (children’s data) explores the birth conditions, access to pediatric health care, previous hospitalizations, recent history of diarrhea and acute respiratory infection, and vaccination history.

The interviews were conducted based on a data collection instrument specially prepared for this study, based on previous experiences of our research group [74,75,76]. The questionnaire was applied with the support of Indigenous Health Agents who work in the communities and/or with the support of the local Indigenous leaders, such as chiefs and teachers.

Responses were recorded on electronic forms with the aid of portable electronic devices (tablets), and there was no use of paper forms. After conducting home visits and interviews, families were invited to participate in a standardized clinical and laboratory evaluation, described in the next section.

### 2.6. Health Assessment

(1) Anthropometric Measurements

All participants had their weight and height recorded. For weight measurement, an electronic scale was used (SECA^®^, model 770, Vogel & Halke, Hamburg, Germany), with a maximum capacity of 150 kg and accuracy of 0.1 kg. Infants had their weight measured on the mother’s lap, using the electronic scale’s mother-baby function. To measure height, a vertical anthropometer or stadiometer of the Alturexata^®^ brand was used (with a dorsal adapter for infantometer and 0.1 cm precision for younger children). Children with special physical needs or suffering from problems in neuro-psychomotor development were excluded from anthropometric analyses. Finally, the body mass index (BMI), expressed in kg/m^2^, for those over 12 years old, and the Z-scores (adjusted for sex and age), for children from 0 to 5 years old, were calculated according to the WHO reference population [77].

(b) Hemoglobin Dosage

The measurement of capillary hemoglobin was assessed using the HemoCue^®^ device (HemoCue^®^, model HB 301-System, Angelholm, Sweden) without the need to collect and store venous blood samples. Hemoglobin levels were classified as normal according to the age group and sex of the participants: (a) children aged 2 to 6 years: between 11.5 to 13.5 g/dL; (b) children from 6 to 12 years old: between 11.5 to 15.5 g/dL; (c) men > 12 years: between 14 to 18 g/dL; (d) women > 12 years: between 12 to 16 g/dL; (e) pregnant women > 11.0 g/dL. Values below the levels indicated above were considered indicators of anemia [78].

(c) Glucose Dosage

Plasma levels of casual blood glucose (measured during the interview, without considering the interval since the last meal and without fasting requirement) were taken from participants over 12 years of age. Was used the Accu-Chek Active^®^ blood glucose monitor (Roche, Indianapolis, IN, USA), auto coded, with photometric biosensor technology (reflectance), and measurement range between 10 to 600 mg/dL, according to the manufacturer’s specifications.

(d) Blood Pressure Measurement

Blood pressure was measured using an automatic pulse blood pressure monitor, Omron Model Hem-631INT (Omron Healthcare INC, Lake Forest, IL, USA). Blood pressure was measured twice, with the device placed on the left wrist of participants over 12 years of age, during the clinical evaluation. Participants remained seated with both feet flat on the floor, with the left forearm resting on the chest’s anterior part, during measurements. The means of the two measures of systolic blood pressure (SBP) and diastolic blood pressure (DBP) were used to classify the participants. According to the Brazilian Guideline for Hypertension of the Brazilian Society of Cardiology [79], hypertension is considered when SBP values are ≥140 mmHg and/or DBP ≥ 90 mmHg. Individuals with an SBP between 130 and 139 and a DBP between 85 and 89 mmHg are considered as prehypertension, as this population has a consistently higher risk of CV disease, coronary artery disease, and stroke than the population with levels between 120 and 129 or 80 and 84 mmHg. For this reason, we included in this study all individuals who had an SBP ≥ 130 and a DBP ≥ 85 mmHg in the high blood pressure group [79].

(e) Prevalence of Sexually Transmitted Diseases

Rapid tests for HIV, hepatitis B and C, and syphilis were performed on indigenous people over 12 years old of both sexes. Following the Ministry of Health’s testing protocol, the HIV tests used were ABON HIV^®^ (Abon Biopharm, Hangzhou, China). In cases with differences in the results, the MEDTESTE HIV^®^ kit (Hangzhou Biotest Biotech Co., Hangzhou, China) was used to resolve disagreements. The hepatitis B test used was BIOCLIN^®^ (Bioclin, Belo Horizonte, MG, Brazil), and for hepatitis C the rapid test from the company ALERE^®^ (BioMérieux, Durham, NC, USA) was used. Finally, for syphilis, the Bio Syphilis kit brand BIOCLIN^®^ (Bioclin, Belo Horizonte, MG, Brazil) was used.

As the Ministry of Health recommended, participants were advised on testing and sexually transmitted diseases before testing. When necessary, counseling was given with results alongside referral to specialized services.

(f) Neurological Evaluation

This component of the study sought to elucidate neurological abnormalities in indigenous older than 12 years, evaluating changes in the Central Nervous System (CNS) and the Peripheral Nervous System (PNS). All participants were submitted to a systemized neurological examination protocol, specially developed for this research, to make the evaluation feasible in adverse conditions in the fieldwork in the villages. The evaluations were conducted by a neurologist (RAAO) familiar with clinical neurological semiology and duly trained to apply the protocol and use the examining instruments.

Abnormalities in the somatosensory, motor, and cognitive function were assessed by the following neurological parameters: static balance and gait assessment; examination of cranial nerves, motricity, somatic sensitivity, and cognitive assessment. The detailed methodology can be accessed elsewhere [80].

(g) Pediatric Evaluation

Indigenous children from 0 to 11 years old were submitted to a pediatric evaluation to analyze immunization coverage and growth curves. The analysis was performed from data on child vaccination registration booklets and booklets for prenatal care. Additionally, we performed an analysis of the nutritional status by weight and height/length measurements (described in the section anthropometric measurements). According to the World Health Organization, the measures of weight and height/length of children under five years old were transformed into Z-scores (adjusted for sex and age), and the weight for age (W/A), height for age (H/A), and body mass index for age (BMI/A) indices were calculated, according to the reference population proposed by the WHO [77].

Moreover, the Denver II Neurodevelopment Screening Test was carried out to evaluate children aged from 0 to 6 years without morphological abnormalities. The test is divided into four areas: (i) personal-social (aspects of the child’s socialization inside and outside the family environment); (ii) fine motor skills (hand-eye coordination, manipulation of small objects, etc.); (iii) language (production of sounds, ability to recognize, understand and use language); and (iv) gross motor skills (body motor control, sitting, walking, jumping and the other movements performed by large muscles). The detailed methodology can be accessed in Hofer et al. [81].

### 2.7. Genetic Polymorphism Analysis

Epithelial cell samples were collected from the oral mucosa to analyze genetic polymorphisms in genes related to mercury metabolism in the human body. The cells were collected using a sterile swab (i.e., disposable cotton swab) and stored in a buffered solution (i.e., a phosphate-buffered saline solution used to prevent changes in the pH and the oral mucosa cells disruption) under refrigeration until arrival at the laboratory. The genomic DNA present in the samples was extracted with an extraction kit (Qiagen Sciences, Germantown, MD, USA), following the procedures recommended by the manufacturer.

The analyses were processed at the Laboratory of Pharmaceutical Science-LAPESF (https://lapesfuezo.wixsite.com/website, accessed on 19 June 2021) of the State University of the West Zone-UEZO, in Rio de Janeiro-RJ, using the polymerase chain reaction technique (PCR) in real-time, as previously described [82,83]. The *TNF-α* −*1031T>C (*rs1799964), −*857 C>T* (rs1799724) and −*308 G>A* (rs1800629), *IL6 −174 G>C* (rs1800795), *ALAD 177 C>G* (rs1800435), *GSTP1 A>G* (rs1695), *VDR Fokl C>T* (rs2228570) and *MMP2 −735 C>T* (rs2285053) polymorphisms were genotyped using a validated TaqMan allelic discrimination assay obtained from Applied Biosystems (C_7514871_10, C_11918223_10, C_7514879_10, C_1839697_20, C_11495146_10, C_3237198_20, C_12060045_20 and C_26734093_20, respectively). In each reaction, two negative (blank) and two positive standardized controls of each genotype were used to guarantee the genotyping quality. The allele frequency and genotype distribution were derived by gene counting and deviations from the Hardy–Weinberg equilibrium (HWE) were assessed by the goodness-of-fit χ^2^ test. The frequencies between the groups were compared using the χ^2^ test or, when appropriate, the Fisher’s exact test.

### 2.8. Determination of Mercury Levels

(a) Hair Samples

Hair samples were collected from all participants, removed close to the scalp in the occipital region with the aid of stainless-steel dissection scissors. The samples were stored in paper envelopes, individually identified, and sent for analysis of total mercury levels (THg) in the Toxicology Laboratory, in the Environment Section of the Evandro Chagas Institute (IEC), in Belém-Pará, Brazil.

In the laboratory, the samples were washed with Extran diluted 100 fold (Merck KGaA, Darmstadt, Germany) to remove any exogenous contamination. After drying, the samples were finely homogenized in glass flasks, prior to weighing. The methodology developed by Akagi [84] involves the steps of chemical opening, wet digestion, and subsequent reduction with SnCl_2_ for quantification of THg in Cold Vapor Atomic Absorption Spectrometer (CVAAS).

Thus, approximately 10 mg of hair was weighed in 50 mL volumetric flasks and subsequently digested with a solution containing 1 mL of deionized water (Milli-Q Milipore^®^), 2 mL of concentrated HNO_3_ and HClO_4_ (1:1), and 5 mL of H_2_SO_4_ on a plate heated at 230 °C for 30 min. About 5 mL of each sample solution was used to quantify THg in the CVAAS Mercury Analyzer Hg-201 (Sanso Seisakusho Co., Nagahama, Japan). To guarantee the Quality Assurance (QA)/Quality Control (QC), for the hair mercury analysis we used the following parameters: (i) the Human Hair Certified Reference Material (IAEA-86), whose average recovery rate was 101% (*n* = 8, recovery ranging from 83.4 to 106.6%) from the International Atomic Energy Agency; (ii) a method blank; (iii) a 6-point calibration curve (concentration ranging from 0.4 to 4 ng/g); and (iv) the relative standard deviation (RSD) of 8.32%. Sample replicates (*n* = 10), whose RSD was 2.49%, were also randomly selected. The detection and quantification limits (LOD/LOQ) obtained were 0.0083 ng/mg and 0.027 ng/mg, respectively. We considered the mercury level of ≥6.0 µg/g in hair samples as a health risk indicator, following the safety dose recognized by WHO [69] and the parameters of previous studies carried out in the Amazon region [20,47,76,85]. This methodology for determining mercury in hair samples was previous described by Akagi [84] and Marinho et al. [20].

(b) Fish Samples

Fish samples were caught at different points at the Tapajós River (4°40′48.33″ S 56°32′35.77″ W; 4°45′21.91″ S 56°27′2.80″ W; 4°42′27.99″ S 56°37′25.62″ W; 4°44′29.18″ S; 56°27′2.79″ W), along the Jamanxin River (4°54′10.83″ S 56°28′2.02″ W; 4°53′24.34″ S; 56°27′20.33″ W) and at its mouth (4°44′56.17″ S 56°26′5.67″ W), with the help of indigenous Munduruku fishermen. A hook or gillnet was used for fishing by the studied communities. The captured fish were identified in the field, photographed, weighed, and measured for total and standard length. Then, about 10 g of muscle tissue was collected from each fish, packed and labeled in plastic bags, and stored in liquid nitrogen to be further analyzed.

Total Hg determination in the fish muscle tissue was obtained using the methodology proposed by Akagi et al. [84], using semi-automatic mercury analyzer equipment, Analyzer Model Hg-201 (Sanso Seisakusho Co., Ltd., Nagahama, Japan) [86]. The detailed methodology can be accessed in Vasconcellos et al. [87].

### 2.9. Health Risk Assessment

The health risk assessment was carried out according to the methodology proposed by WHO [88]. Four different scenarios of methylmercury exposure were built from the data collected for a counterfactual analysis: (i) for fish consumed in the rainy season; (ii) for fish consumed in the dry season; (iii) current, considering an empirical method to estimate fish consumption; and (iv) critical, regarding the consumption from the 95th percentile of mercury concentrations in piscivorous and non-piscivorous species. The risk ratio was calculated from the ratio between “the estimated MeHg intake” in the four different scenarios and the reference doses proposed by FAO/WHO [25] and U.S. EPA [26]. The detailed methodology can be accessed in Vasconcellos et al. [87].

### 2.10. Statistical Analysis

We carried out a descriptive analysis with the characterization of the study population, including household characteristics (family monthly income, infrastructure of the house, and sanitation) as well as sociodemographic characteristics (gender, age group, marital status, schooling), and clinical data of the study population (hemoglobin, glucose and blood pressure levels, as well as body mass index). Moreover, we presented the hair mercury levels of the participants according to the sociodemographic and clinical studied variables.

In order to contrast clinical and sociodemographic variables with hair mercury exposure ≥6.0 µg/g versus hair mercury exposure <6.0 µg/g, we used Pearson’s Chi-squared test or Fisher’s exact test. We also used the Kruskal–Wallis test to evaluate the differences in Hg levels between villages. To compare the hemoglobin, glucose, and blood pressure mean levels, as well as height, weight, and body mass index between the participants in the mercury exposure groups ≥6.0 µg/g x ≤ 6.0 µg/g, we used the F-Test.

To estimate the prevalence of human mercury exposure in the study area, we considered the number of people who presented mercury levels ≥6.0 µg/g as the numerator, and the denominator as the population sampled in the study region, according to the villages: *Sawré Muybu*, *Poxo Muybu,* and *Sawré Aboy*.

To explore factors associated with mercury exposure, we used the logistic regression model with conditional Prevalence Ratio (PR). In this study, PR was used as an association measure with the respective 95% confidence intervals. After the initial crude analysis, the variables with a significant association with the hair-Hg level were further adjusted in this model. According to Bastos et al. [89], the logistic regression model provides a better estimate of the prevalence ratios, representing a more significant measure of effects for cross-sectional studies. The 95% confidence intervals were obtained using the delta method and clustered bootstrap. A significance level of 5% (*p* < 0.05) was considered for all statistical tests used. The data were analyzed using the free statistical software R version 3.6.3 (http://www.r-project.org, accessed on 31 March 2021) and the package “prLogistic” [90].

## 3. Results

### 3.1. Principal Research Results

#### 3.1.1. Sociodemographic Characterization

During fieldwork, the research team visited 35 households in the Sawré Muybu Indigenous Land: 8 in Poxo Muybu village, 7 in Sawré Aboy village, and 20 in Sawré Muybu village (Table 1).

In most families, regular incomes from salaries and or social benefits from the government were reported. The average monthly income of families was USD 294.10 ranging from USD 201.0 to USD 414.5, with averages in *Poxo Muybu* slightly higher and *Sawré Aboy* somewhat lower. Concerning physical structure, approximately 60.0% of households had dry straw-covered roofs, 65.7% had wooden or brick walls, and 80.0% had dirt floors (Table 1).

There was no bathroom for exclusive family use in any of the houses visited. Most of the heads of households reported that their family members use a collective cesspool available in the community. The vast majority of interviewers (91.4%) reported that the water consumed by the families came from rivers/streams, and 68.6% reported there is no type of water treatment in the villages. Only three homes from *Sawré Muybu* village reported an artesian well as water supply (Table 1).

Regarding the length of residence, in the *Sawré Muybu* village, most families (70.0%) had been settled for more than five years, while families had been set in the *Sawré Aboy* and *Poxo Muybu* villages up their homes within the last three years. In *Sawré Muybu*, there were between one and two adults in most households (75.0%), while in *Poxo Muybu*, half of the households were composed of more than four adults. In most of the households visited, there were between two and three children under five years old.

Two hundred participants were interviewed and clinically evaluated, 66 from *Poxo Muybu*, 40 from *Sawré Aboy,* and 94 from the *Sawré Muybu* village. The study population was predominantly young, with an average age of 18 (median: 14; IQR: 6, 14, and 25 years old). Adults over 45 years old represented only 7.0% of all study population. In general, the presence of female participants was more evident, except for *Sawré Aboy* village, where 48.0% were women (Table 2).

Almost three-quarters (71%) of the participants had only 1 to 9 years of schooling, with more years spent in education (10 years or more) observed in the *Poxo Muybu* village (21%). The most frequent marital status was married (39% of the participants), being more frequent in the *Sawré Muybu* village (41%) (Table 2).

Considering the principal occupational activities, participants above 12 years old (*n* = 112) reported working as a farmer (38.7%), carrying out activities at home (37.8%), student (32.4%), fisherman (18.9%), teacher (10.8%), extractors of natural forest products/hunter (9.9%), bricklayer (5.4%), retired (3.6%). Only one participant designated themself as unemployed. It is worth remembering that participants can do more than one activity simultaneously.

Ten men (seven from *Sawré Muybu*, two from *Poxo Muybu*, and one from *Sawré Aboy*) aged 26 to 67 years (mean 38 years) reported working previously in mining activities. Half of them reported having worked at mining for less than five years. Seven participants had between 1 and 9 years of schooling, and three had more than ten years. The median mercury level in the hair samples of these participants was 6.8 µg/g (mean: 8.8; standard deviation: 4.9; range: 3.5 to 18.7), and 6 of them had levels above 6.0 µg/g.

Interviews regarding diet revealed that almost all families (96%) eat fish frequently (average three times per week). Moreover, the study participants related that fish consumption varies according to the season (dry or rainy) and fish availability in the region’s rivers. During the rainy season, the most consumed fish species are *aracu* (family *Anostomidae*), *surubim* (*Pseudoplatystoma* spp.), *barbado* (*Pinirampus pirinampu*), *matrincha* (*Brycon* spp.), *tucunaré* (*Cichla* spp.), and *caratinga* (*Geophagus* spp.). During the dry season, the most consumed species are *caratinga*, *curimata* (*Prochilodus nigricans*), *surubim*, *pacu* (family *Serrasalmidae*), *barbado*, *mandiá* (*Pimelodina flavipinnis*), *aracu*, *aruana* (*Osteoglossum bicirrhosum*), *piranha* (family *Serrasalmidae*), and *matrincha*.

#### 3.1.2. Health Assessment

Regarding the self-reported morbidities, in the month before our team’s visit, we highlight the high frequency of flu/colds, fever, and diarrhea that were mentioned by 44.8%, 17.2%, and 12.0% of the participants, respectively.

Considering previous hospitalizations, 69 individuals (35.0% of the study population) reported at least one hospitalization throughout their lives. The highest frequencies were reported in *Sawré Muybu* (53.2%) and *Sawré Aboy* (35.0%) villages. While in *Poxo Muybu*, only 10.6% of the participants reported previous hospitalization. Sixty-six participants (31.0% of all interviews) reported at least one previous treatment for Malaria. The highest proportions of previous treatment for Malaria were identified in *Sawré Muybu* (41.5%) and *Sawré Aboy* (32.5%) villages. In *Poxo Muybu*, 21.2% of participants had previous treatment for Malaria.

There were 53 women of childbearing age in the study area. Two-thirds of women reported having given birth at least once. For them, the number of completed pregnancies ranged from 1 to 14, and almost 70% reported they have less than seven children. Five women were pregnant, and 17 reported were breastfeeding. For all women within the study population, including those aged above 49 years old, three-quarters having given birth at least once, one-fifth reported previous spontaneous abortion, while almost half mentioned at least one fetal death. For more details, see Kempton et al. [91].

#### 3.1.3. Anthropometric Measurements

The body mass index (BMI) analysis shows that 63.5% of the adults are within normal ranges of weight and height (BMI range 18.5 to 24.9 Kg/m^2^). However, the analysis by village reveals that the population of *Sawré Muybu* represents most cases of being overweight (BMI range 25.0 to 29.9 kg/m^2^), while *Poxo Muybu* reported the highest rate of obesity (BMI ≥ 30.0 km/m^2^) (Table 2).

#### 3.1.4. Hemoglobin Levels

The analysis of hemoglobin levels reveals that one-third (33.0%) of the participants had anemia. The prevalence varies between the villages, with the most challenging scenario was observed in the *Sawré Aboy* village, where 48% of the participants had anemia (Table 2).

Anemia was present in 21.1% of the children under five years old, more evident among children aged 6 to 12 months, whose 66.7% had hemoglobin levels under 11.5 g/dL. This index suggests a prominent micronutrient deficit when exclusive breastfeeding is interrupted. Despite the variations of the hemoglobin levels between the studied villages, there were no associations with Hg levels.

#### 3.1.5. Glucose Levels

Due to logistical and operational problems, the dosage of glucose levels was carried out only among 36 adults living in the *Poxo Muybu* village. Plasma levels of casual blood glucose ranged from 94.0 to 197.0 mg/dL (median: 110.0 mg/dL; standard deviation: 18.4 mg/dL), not exceeding the 200 mg/dL limit established as a reference to indicate a diagnostic suspicion of diabetes mellitus. Moreover, the glucose levels were not associated with Hg levels.

#### 3.1.6. Blood Pressure Levels

Looking at the blood pressure levels of those over 12 years old, we observed that only 13 participants presented hypertension or pre-hypertension, understood as systolic blood pressure (SBP) above 130 mmHg, and diastolic blood pressure (DBP) above 85 mmHg. Thus, on average, the prevalence of hypertension was 11.3%. Again, there was variation between the villages, and the problem was more prominent in the *Sawré Muybu* village, where 14.5% of the participants have high blood pressure levels. In addition, the highest levels of systolic blood pressure and diastolic blood pressure have been associated with hair mercury levels above 6.0 µg/g (F-Test: 4.157, *p*-value: 0.044; and F-Test: 4.363, *p*-value: 0.039, for SBP and DBP, respectively).

#### 3.1.7. Sexually Transmitted Diseases

One hundred and twenty-three rapid tests were carried out to investigate hepatitis B infection. A 10-year-old girl living in the *Poxo Muybu* village presented a positive result (0.8%). Following the Brazilian Ministry of Health guidelines, our team repeated the test, and in the end, the test proved to be non-reactive. One hundred and twenty rapid tests were carried out to investigate infection by the hepatitis C virus. All of them proved to be non-reactive.

One hundred and twenty-three rapid HIV tests were carried out. Of these, five showed positive results in the first test (4.1%): a 19-year-old woman, a 23-year-old man, and a 39-year-old man, living in the *Sawré Aboy* village, and two men (one is 27 and the other is 30 years old), living in *Sawré Muybu* village. Following the Brazilian Ministry of Health guidelines, our team repeated the tests twice, using kits from different manufacturers. In the end, only the 19-year-old woman from the *Sawré Aboy* village remained undetermined. We referred all participants who presented problems in the tests to the Unified Health System (SUS) for follow-up. Moreover, 123 tests were carried out to investigate infection of Treponema pallidum, the causative agent of syphilis, all of which proved to be non-reactive.

#### 3.1.8. Neurological Evaluation

Somatosensory, motor, and cognitive abnormalities were investigated in indigenous over 12 years old in the three studied villages. The principal abnormalities—which correspond to somatosensory dysfunctions and alterations in verbal fluency—were more prevalent in participants with mercury exposure levels above 10 µg/g who live in the *Sawré Aboy* and *Sawré Muybu* villages. Moreover, participants with higher levels of mercury exposure presented the highest prevalence of cognitive development deficits, in addition to motor signs such as cerebellar ataxia. These findings suggest a worsened motor and cognitive function resulting from neurotoxicity due to chronic Hg exposure. Further details can be seen in Oliveira et al. [80].

#### 3.1.9. Pediatric Evaluation

The pediatric evaluation revealed that less than half of the children had a complete vaccination schedule. Nine of the 55 eligible children under six years old showed problems in the neurodevelopment tests. The main observed problems include limitations in the language, fine motor and gross motor skills, and personal-social components of the test. Among the nine children who presented problems in the neurodevelopment tests, the average mercury levels in hair samples were 7.34 µg/g (median: 6.2; range from 2.4 to 19.6; standard deviation: 5.4 µg/g). In contrast, the average mercury levels for all children under 11 years were 6.9 (median: 5.5; range from 1.4 to 23.9; standard deviation: 4.8 µg/g).

We measured the weight and height/length of 42 children under 5 years old. The analysis of the Z-scores (adjusted for sex and age) revealed that on average, 26.2% presented with stunting (Z-scores < −2.0 to height for age), and 7.1% were underweight (Z-scores < −2.0 to weight for age), while 16.0% were at risk of being overweight (Z-scores > 1.0 to weight for height. Additional information can be seen in Hofer et al. [81].

#### 3.1.10. Genetic Polymorphism Analysis

Polymorphisms in genes involved in mercury toxicity were analyzed and the efficiency of the genotyping ranged between 96.5% to 100% (192 participants). The distribution of studied polymorphisms was in Hardy–Weinberg equilibrium in the overall study population. The minor allele frequencies (MAF) of the *TNF*-α (rs1799964, rs1799724 and rs1800629), *IL*-6 (rs1800795), *ALAD* (rs1800435), *GSTP1* (rs1695), *VDR* (rs2228570) and *MMP2* (rs2285053) in the population was 13.1%, 28.9%, 2.3%, 0.3%, 0.5%, 35.9%, 34.2%, and 12.4%, respectively. Table 3 shows the genotype distribution of the eight polymorphisms among the three villages. There were significant differences (*p*-value < 0.05) in the genotypic distribution among the three villages for the *TNF*-α rs1799964 and rs1800629, *GSTP1* rs1695, *VDR* rs2228570 and *MMP2* rs2285053 polymorphisms.

Each gene/polymorphism will be evaluated in the context of chronic mercury exposure and on the health situation of the Munduruku population in further publications. Firstly, regarding *ALAD* (rs1800435) polymorphism, two individuals from the *Sawré Muybu* village were heterozygous and presented high mercury concentrations, as well as severe symptoms of chronic mercury exposure. For more details, see Perini et al. [92].

### 3.2. Determination of Mercury Levels

#### 3.2.1. Hair Samples

**Figure 2:**
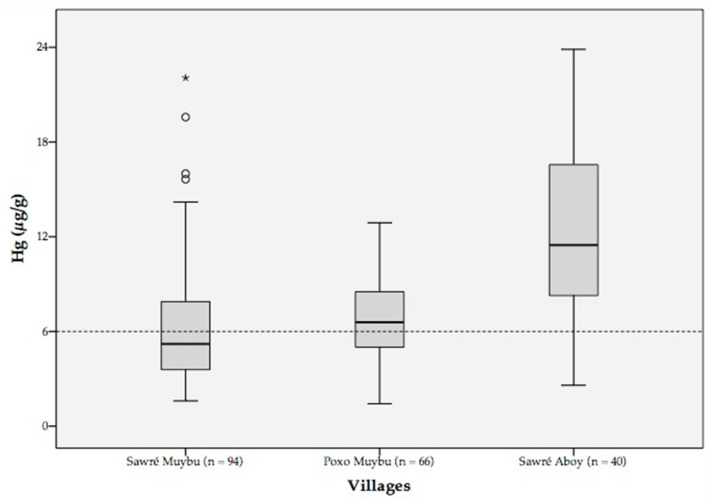
Boxplot with total hair mercury concentrations (µg/g), according to the investigated villages. *Sawré Muybu* Indigenous Land, Pará, Amazon, Brazil, 2019. Obs: The dashed line corresponds to the safe Hg exposure level adopted in this study (6.0 µg/g). The circles correspond to the cases that showed hair mercury levels that surpassed the superior limit of the interquartile (IQR). The asterisk corresponds to an outlier case in the *Sawré Muybu* village from one indigenous who presented a hair mercury level equal to 22.1 µg/g.

Hair samples analysis showed mercury levels ranging from 1.42 to 23.9 µg/g. The median mercury level for the entire population was 6.6 µg/g (mean: 7.7; standard deviation: 4.5, Q1: 4.5; Q3: 9.5). There were statistically significant variations between the villages: *Sawré Muybu* (5.2 µg/g), *Poxo Muybu* (6.6 µg/g), and *Sawré Aboy* (11.5 µg/g) (Kruskal–Wallis = 42.2; *p*-value < 0.001); and between adults and children (Kruskal–Wallis = 10.4; *p*-value = 0.001) (Table 4, Figure 2).

On average, the prevalence of exposure above 6.0 µg/g for all villages was 57.9%. In the *Sawré Muybu* village, we observed the lowest prevalence (42.9%), while in the *Sawré Aboy* village, was reported the highest (87.5%).

In addition, the mercury average levels among children (5.9 μg/g) were slightly lower than adults (7.3 and 6.3 μg/g for men and women, respectively), in the *Sawré Muybu* village. It is worth remembering that the highest mercury level (22.1 μg/g), in the *Sawré Muybu*, was reported in a 5-year-old girl (Table 4).

Within the *Poxo Muybu* village, the prevalence of mercury exposure above 6.0 µg/g was 60.6%. The average mercury levels among children (5.9 μg/g) and adults (7.1 and 7.6 μg/g for men and women, respectively) were similar when compared to *Sawré Muybu* (Table 4).

In contrast, in the *Sawré Aboy* village, the prevalence of mercury exposure reached 87.5% (Table 4). Moreover, the average mercury levels among children (11.0 μg/g) and adults (13.6 and 12.1 μg/g for men and women, respectively) were almost two-fold, compared to those observed in *Sawré Muybu*. It is worth noting that the highest level of mercury in our study population (23.9 μg/g) was registered in a 10-year-old boy living in *Sawré Aboy* (Table 4).

Considering the sociodemographic characteristics among indigenous older than 12 years, only “income” and “village of residence” were associated with the prevalence of mercury exposure ≥ 6.0 µg/g (*p*-values equal to 0.042 and 0.002, respectively) (Table 5). This means that the prevalence of hair mercury levels above 6.0 ug/g was higher among individuals who declared had no income (73.1%), in contrast to those who had income (26.7%). Similarly, the prevalence of hair mercury levels above 6.0 ug/g was higher among individuals living in the *Sawré Aboy* village (73.7%), compared to they were living in *Poxo Muybu* (71.1%) and *Sawré Muybu* (52.8%) (*p*-value: 0.002). The other variables did not show statistically significant differences.

Regarding the clinical characteristics, only indigenous with high blood pressure levels, classified as “hypertension”, showed a statistically significant association (*p*-value < 0.20) with mercury levels ≥ 6.0 ug/g. In this case, 92.3% of the indigenous older than 12 years diagnosed with hypertension have mercury levels in hair samples greater than or equal to 6.0 µg/g (Table 5).

Concerning the women of childbearing age, only the variable “pregnancy” showed a statistically significant association with the prevalence of contamination ≥ 6.0 µg/g at the level of 10% (*p*-value, 0.067). All pregnant investigated women showed hair mercury levels above 6.0 µg/g. The variables “number of children” and “breastfeeding” showed no significant association (Table 6).

Table 7 shows the comparison between the mean values of the variables that make up the clinical characterization of individuals older than 12 years. The means of the “SBP” and “DBP” variables were higher in the group with mercury exposure ≥ 6.0 µg/g, with *p*-values equal to 0.044 and 0.039, respectively. The other tested variables did not reveal any differences between the groups, considering the mercury exposure groups.

The logistic regression model applied to all adult participants (men and women ≥ 12 years old) pointed out that the prevalence of mercury exposure ≥ 6.0 µg/g was significantly higher among participants living in the *Sawré Aboy* village (adjusted PR: 1.8; 95% CI: 1.3–2.3; *p*-value: 0.001), among participants who had no regular income (adjusted PR: 1.3; 95% CI: 1.0–1.8; *p*-value: 0.031) and among participants who have had hypertension on clinical evaluation (adjusted PR: 1.6; 95% CI: 1.3–2.1; *p*-value: 0.01).

For the women of childbearing age, the logistic regression model revealed that the prevalence of mercury exposure ≥6.0 µg/g was 2.5 times higher in women living in the *Sawré Aboy* village (adjusted PR: 2.5; 95% CI: 1.4–4.4; *p*-value: 0.001), 1.9 times higher in women living in the *Poxo Muybu* village (adjusted PR: 1.9; 95% CI: 1.0–3.4; *p*-value: 0.044), 1.9 times higher among women with hypertension (adjusted PR: 1.9; 95% CI: 1.2–3.3; *p*-value: 0.012) and 1.5 times higher among pregnant women (adjusted PR: 1.5; 95% CI: 1.0–2.1; *p*-value: 0.029) (Table 8).

#### 3.2.2. Fish Samples

In total, 88 fish specimens were captured, distributed in 17 species and 4 trophic levels. Three piscivorous species showed average mercury levels above 0.5 µg/g. The Hg-biomagnification in the fish trophic chain was confirmed, as there was a significant difference in the THg concentration in muscle tissues between trophic levels.

The highest level of mercury in muscle samples (1.95 µg/g) was observed in the *Serrasalmus rhombeus* (also known as *piranha preta*), a fish located on the top of the food chain. All results on the capture and determination of mercury in fish samples can be accessed in Vasconcellos et al. [87].

### 3.3. Health Risk Assessment

With dietary patterns in mind, estimates of fish mercury levels place daily methylmercury consumption above the safe limits recommended by the FAO/WHO [25], and the U.S.EPA [26] in all scenarios and in all studied population strata (i.e., children, childbearing women, and men).

Estimates of Hg ingestion, performed here, indicated that the daily methylmercury intake surpasses up to 11 times the FAO/WHO [25] and exceeds the U.S. EPA [26] reference dose from 3- to 25-fold. In all scenarios analyzed (dry season, rainy season, current, and critical), the risk ratio estimates were above 1.0, meaning that the studied Munduruku villages are at severe risk of harm due to ingestion of mercury-contaminated fish. Detailed findings can be accessed in Vasconcellos et al. [87].

## 4. Discussion

This is the first time a research group has prepared a multidisciplinary and inter-institutional study to meet the demand of an indigenous association in order to clarify the consequences and assess the impacts of illegal mining in their territory.

Through this investigation, it was possible to better understand the impact and magnitude of decades of illegal economic activities within the *Sawré Muybu* Indigenous Land and adjacent areas, with emphasis on direct and indirect health impacts caused by the ASGM. Without exception, we detected relevant mercury levels in all hair samples analyzed, including adults, elderly, men, childbearing women, and children. In fact, all of the indigenous Munduruku who participated in the study showed hair mercury levels above the safe limits established by the most varied international health agencies [25,26,88]. Considering the safety parameter adopted by the present study (i.e., 6 µg Hg/g in hair), on average, 6 out of 10 participants had mercury levels above this limit. However, human mercury exposure was not homogeneous in the three villages investigated and was directly associated with the intensity and extent of mining activity in each location. The *Sawré Aboy* village, located downstream from the Jamanxin River, was the closest to the mining activities (see Figure 1). In its surroundings, the environmental effects of the mining activity were easily observed. Since there were many floats for gold extracting from the river sediment, the river’s water quality was compromised, and deforestation areas in the riverbanks for the excavation could be noted. In that village, 9 out of 10 participants had Hg exposure levels greater than 6.0 µg/g, with median levels of 11.5 µg/g. The second village most impacted by gold mining was *Poxo Muybu*. According to data published by the RAISG [21], there are many illegal mining points in the vicinity of that village (Figure 1). The median levels of mercury in hair samples collected from residents of that village reflected the intensity of mining activity in that area (6.6 µg/g). Finally, the least impacted village by mining was *Sawré Muybu*, and as a consequence, its residents showed median levels of mercury in hair equal to 5.2 µg/g. In order to support these findings, the abovementioned variations seemed not to be random, once they were statistically significant.

Mercury has been a valuable metal in various industrial processes, particularly in gold mining activities and especially in developing countries surrounding the Amazon basin. This apparent economic benefit is insignificant compared to its severe environmental and neurotoxicological health impact [4,5,6,15,16,17,18,19,20,93]. The present study provides evidence that communities living in the Tapajos River basin in the Munduruku indigenous territories have a mean level of Hg exposure surpassing the safety limit (6.0 μg/g) recognized by the World Health Organization [69].

It is essential to clarify that health agencies do not define safe levels of mercury exposure from concentrations detected in hair samples or other exposure biomarkers. In fact, the limits recommended by the health agencies consist of a definition of a maximum methylmercury intake dose (daily or weekly) that does not cause observable health effects. However, as there is a well-defined correlation between MeHg intake and levels detected in hair and blood, it is possible to establish correspondences. Remarkably, the intake recommendations vary significantly from one agency to another, and that occurs because the data used for the estimations are collected from different studies, including different populations and regions of the planet.

The present study used 6.0 µg/g of mercury in hair samples as a threshold for toxic effects, according to other research studies developed in the Amazon [20,47,76,85]. Kjellstrom et al. [94,95] and Crump et al. [96] adopted this concentration during the New Zealand cohort study to identify women with high mercury exposure.

This hair mercury level is derived from calculations for safe intake defined in 1972 by the Joint FAO/WHO Expert Committee on Food Additives (JECFA) [97], which were ratified in 1989 [98] and 2000 [99]. The basis for these estimates was the poisoning episodes of Minamata and Niigata, in Japan, that occurred in the 1950s. From the health endpoints related to poisoning, the JEFCA established a Provisional Tolerable Weekly Intake (PTWI) of 3.3 µg/kg bw/week as a safe intake dose (that corresponds to 6.0 µg MeHg/g in hair).

With the same purpose, the U.S. EPA [26] defined the reference dose (RfD) of 0.1 µg/kg bw/day based on studies carried out in Iraq. This dose was revised from the Faroes Island cohort study’s findings and confirmed in the year 2000. In this case, the hair mercury levels correspond to 1.0 µg MeHg/g. Nowadays, the FAO/WHO [25] recommends doses of 1.6 µg/kg bw/week for vulnerable groups (such as women of childbearing age and pregnant), which correspond to mercury hair levels of 2.3 µg/g. In contrast, for the general population, the PTWI recommended is 3.2 µg/kg bw/week, which corresponds to a hair level of 4.5 µg/g [25].

The variety of safe limits reveals the challenges in developing global monitoring and surveillance instruments to identify populations at risk of mercury exposure. It occurs because the proposed doses result from observing the health mercury effects in different population groups and from very different exposure scenarios (e.g., chronic exposure observed in cohort studies and poisoning episodes in Iraq and Japan). Besides that, this limitation reinforces the need to develop longitudinal studies in native populations from the Amazon, such as indigenous and riverine populations, that consume large amounts of fish. These groups share a unique exposure scenario worldwide, where there is a combination of natural mercury present in the soil and different anthropic actions that increase the supply of mercury to the Amazon ecosystem, which then affect the biogeochemical cycle of this element, such as gold mining, the construction of dams, hydroelectric plants and forest fires [27,48,50].

In our study, the logistic regression model revealed that sociodemographic variables such as “income” and “village”; clinical variables, such as “hypertension,” as well as pregnancy in women of childbearing age showed a significant association with hair mercury levels above 6.0 µg/g. Focusing on the findings related to “income,” the prevalence of mercury exposure above 6.0 µg/g was 30% higher among indigenous who had no source of regular income, for example, salary, cash transfer, pension, retirement, among others. The absence of regular income to some families can impair the access to other protein sources in the diet besides fish, such as meat, chicken, pork, and other products that came from markets in the nearest municipalities. Consequently, these families potentially increase their ingestion of mercury-contaminated fish and become more prone to illness due to contamination, worsening the scenario of social and environmental vulnerability in the villages. Theoretically, the consumption of other protein sources would ensure a more diversified diet as well as less dependence on the protein obtained from fish consumption. Therefore, the lowest hair mercury levels would be observed in individuals who have a regular income.

Another important finding revealed that the indigenous living in the *Sawré Aboy* village had had 80% more prone to have mercury levels above 6.0 µg/g. When we analyzed the women of childbearing age who live in that village, the prevalence of mercury levels above 6.0 µg/g is 2.5-fold higher. As previously mentioned, in that community, the proximity to the ASGM is more significant than in other villages, contributing to the detection of higher levels of contamination in humans.

Among the studied clinical variables, it was observed that the prevalence of mercury exposure above 6.0 µg/g was 1.6 times higher in adults (including men and women) who presented with hypertension. In women of childbearing age, the prevalence of mercury exposure above 6.0 µg/g was 1.9-fold higher in women with hypertension. Different authors have studied the association between mercury exposure and the incidence of cardiovascular diseases in different contexts [100,101,102,103,104,105]. Fillion et al. [106] investigated the association between mercury exposure with hypertension in riverside dwellers living on the banks of the Tapajós River. The mentioned study enrolled 250 people from six different communities and used a linear regression model, which pointed out a significant association between mercury levels above 10 µg/g and an increase of systolic blood pressure levels in adults (Odds Ratio: 2.91; CI95%: 1.26–7.28), in harmony with our findings.

On the other hand, Dórea et al. [68] did not reported a significant relationship between mercuric exposure and hypertension in an investigation carried out among Kayabi and Munduruku indigenous peoples, 15 years ago. We might suppose that environmental contamination and mercury exposure have worsened in that region over the last two decades, putting the local population at a new risk rate.

In addition, these indigenous communities maintain their cultural and linguistic distinctiveness with substantial genetic distance from other non-indigenous populations. So, it is essential to verify the genetic profile ascribed to inter-populational diversity because data on genetic polymorphisms associated with mercury toxicokinetics and toxicodynamics cannot be extrapolated from other populations. In this study, we identified two individuals with the *ALAD* gene polymorphism which has previously been associated with low enzyme activity and, consequently, with high mercury levels in blood samples [107]. Detailed findings can be found in Perini et al. [92]. Moreover, indigenous people keep consistently underrepresented in genetics databases [82].

There is a long and ongoing history of gold mining activities in the Tapajos River basin and its impacts are well documented [5,13,14,41,43,63,64,66,67,108,109,110]. It is widely known that artisanal gold mining activities are responsible for emitting 200 metric tons of mercury annually; approximately 27% of global emissions are discarded annually without any control [111,112], constituting a potential risk to human health and the environment. It is important to note that, in general, the Munduruku people living in their traditional territories are not involved in gold mining activities, and yet they are victims of this controversial and doubtful development process, suffering the consequences of mercury contamination.

As well as being described by Khoury et al. [16], Santos Freitas et al. [17], Domingues et al. [18], Pinheiro et al. [19], Marinho et al. [20], Vega et al. [76], Barbieri and Gardon [113], our findings suggest that the proximity of the investigated communities with the gold mining activities is related to higher levels of methylmercury in hair samples. The comparative analyses performed on the communities revealed that in the *Sawré Aboy* village, which is located closer to gold mining operations, the greater hair Hg concentrations were reported. According to the WHO [88], human hair is an excellent biomarker for MeHg exposure, because MeHg deposited in the hair remains stable for a long time.

In the Amazon, hair-Hg levels in indigenous communities vary considerably. For instance, the mean Hg concentration in the hair of Munduruku people from Teles Pires (Pará, Brazil) was 3.4 μg/g [64], whereas, in ethnic groups from 19 villages at the Yanomami reserve, these varied between 0.4 and 22.1 μg/g (*n* = 239) [76]. The mean of the T-Hg value reported here (7.7 μg/g) was below that registered for indigenous communities living at the upper Tapajos River basin (14.7 μg/g) [67]. Nevertheless, it was above those found in other Amazonian municipalities where artisanal gold extraction has been established as the primary source of Hg-contamination, such as Belmont (Rondônia) (2.71 μg/g), Cunia (Rondônia) (0.9 μg/g) [114], Novo Airão (Amazonas) (5.67 μg/g) [115], Porto Velho (Rondônia) (0.60 μg/g) [116], and Manaus (Amazonas) (1.93 μg/g) [117].

There is a well-established relationship between human mercury exposure and fish consumption, not only in our study area but also in other parts of the Amazon region [37]. Amazon fish are the most important protein source for the local riverine populations, and fishing is a significant and traditional economic activity throughout the region [118,119]. Traditional communities in this study are likely exposed through the consumption of contaminated fish from the Tapajos River, based on extensive prior evidence of fish as the primary source of human MeHg exposure [41,63,64,66,87].

In addition to high levels of mercury exposure, our study revealed that families live in vulnerable conditions, since the average monthly income does not exceed USD 300.00, and most of it comes from social benefits. Households have a precarious structure, with no bathrooms for the families’ exclusive use, nor potable water for people’s consumption. The level of education is also low, there are not enough jobs available, nor sustainable development projects, and most adults work in informal or subsistence activities. Even some adults end up being enticed to work in mining activities due to an absolute lack of work opportunities.

The precarious scenario experienced in the villages results in other health challenges, which can be observed in the high rates of illness such as diarrhea, influenza, and Malaria, high rates of hospital admissions, and the high number of abortions and fetal deaths, revealing gaps in health services. Additionally, the lack of sanitation plays a significant role in nutritional deviations and in the high rates of anemia recorded in children under five years old [120,121,122,123].

Despite the illustrative findings of this investigation, it is necessary to reflect on some limitations. Our first challenge was bearing in mind the diversity of approaches on chronic mercury exposure in the Amazon territories. We start from a biomedical perspective with a focus on neurological and pediatric evaluations, including the estimation of the burden of infectious disease as well as non-communicable diseases and the analysis of genetic polymorphisms, while considering the fish ecology in the region, and the social context in which the illegal mining activities are performed in that territory. In all approaches, we thought of the appropriate statistical treatment to the cross-sectional source data.

After that, we were faced with the challenge of choosing validated and simplified data collection instruments for use in fieldwork conditions and how these instruments would be used in a differentiated sociocultural context. In this sense, we cannot fail to mention the difficulties faced in conducting extensive interviews with families, who in some situations did not speak Portuguese fluently, requiring the presence of interpreters.

In addition, we handled the challenge of applying child neurodevelopment tests, such as Denver II [124], which has been validated for use in urban contexts in Brazil, therefore, requiring some adaptations from researchers, especially in the language component.

It was also complex to carry out neurological evaluations in adults. In general, other authors value complaints and symptoms reported by participants to assess the harm caused by mercury contamination in adults [88,125]. Our study chose to prioritize the clinical evaluation directed in elements already considered in the literature and focus on identifying signs of somatosensory, motor, and cognitive impairment in young people and adults. Detailed results of this approach can be reached in Oliveira et al. [80].

Furthermore, due to the limited time of contact with the communities and the linguistic difficulties already mentioned, we could not accurately assess the exact amount of fish consumed nor characterize other elements of the families’ diet. To address part of this limitation, we developed an empirical method to estimate household fish consumption, found in the manuscript lead by Vasconcellos et al. [87].

Our study covered only three villages in the region of the Middle-Tapajós River, including a total of 200 participants. The entire Munduruku population is estimated at approximately 12,000 people, distributed in more than 120 villages, primarily concentrated in the region of the upper Tapajós River, where more intensive illegal mining activity is concentrated. Therefore, it is possible that despite robust estimates, our findings are underestimated and do not reveal the true impact on the majority of the Munduruku people from mercury exposure.

Finally, our team could not collect longitudinal data to assess possible changes in the health situation over time (for example, related to seasonality, typical in the Amazon region). Given the limitations mentioned above, it is impossible to make more robust causal inferences about mercury exposure in the region. Therefore, more in-depth studies are needed, with a longitudinal approach, focusing mainly on pregnant women and children under five years old. In our opinion, this is the only way to measure the actual extension of health impacts related to chronic mercury exposure in traditional communities in the Amazon.

On the other hand, this study’s strengths lie in the census of indigenous populations living in the three studied villages, and the use of prevalence ratios in the multivariate analyses, making a sound basis for planning public health interventions.

## 5. Conclusions

In conclusion, the time spent in the communities, the observation of the environmental impacts resulting from illegal mining, and the analysis of the collected data in the fieldwork allows us to make some reflections and therefore propose the following recommendations:

(a) The immediate interruption of illegal mining activities and cessation of invasion of traditional and protected lands of the Amazon.
(b) In parallel, to develop a national plan to discontinue mercury use in artisanal mining to achieve the goals of the Minamata Convention on Mercury.
(c) To develop a risk management plan (RMP) for populations chronically exposed to mercury. The plan should contain a set of integrated guidelines and actions, such as:
  (i) to expand the monitoring of mercury levels in fish consumed not only in traditional territories but also in urban areas of the Amazon.
  (ii) to develop educational material for the population of the affected areas, containing clear information on safe fish consumption, respecting cultural aspects related to different ethnic groups.
  (iii) to include testing of mercury levels in hair samples in the routine of the prenatal care program and in the program for monitoring child growth and development, in the Brazilian Unified Health System (SUS).
  (iv) to develop a Basic Care Protocol for Contaminated People to be equally incorporated into the SUS.
  (v) to promote a research and scientific development program to carry out more in-depth studies to enhance the knowledge about the health impacts of Amazon populations chronically exposed to mercury.
  (vi) to formulate public policies to create sustainable economic alternatives for indigenous communities affected by illegal mining in order to guarantee food security, sovereignty, and respect for ancestral traditions.

Lastly, as a signatory of the 2013 Minamata Convention, the Brazilian Government should commit to combatting environmental contamination and exposure to mercury. It is of particular importance given that, currently, the ASGM is the main responsible for increased levels of mercury in the Amazonian environment. Additionally, the ASGM has been promoted conflicts and attacks within these traditional territories. Summing up, the indigenous populations are looking to the Brazilian authorities to ban and control gold mining in their sacred territories.

## Figures and Tables

**Table 1:** Household characteristics, according to family monthly income, physical structure (flooring, walls, roofing) and sanitation, *Sawré Muybu* Indigenous Land, Pará, Amazon, Brazil, 2019.

Characteristics	Overall *n* = 35	Village		
		*Poxo Muybu* *n* = 8	*Sawre Aboy* *n* = 7	*Sawre Muybu* *n* = 20
Family Monthly income (USD) Median (Q1, Q3)	294.1 (201.0–414.5)	355.7 (274.5–728.4)	235.3 (203.9–485.3)	284.3 (166.7–382.0)
Regular salary				
Yes	24 (68.6%)	6 (75.0%)	6 (85.7%)	14 (75.0%)
No	11 (31.4%)	2 (25.0%)	1 (14.3%)	6 (25.0%)
Regular cash transfer				
Yes	24 (68.6%)	6 (75.0%)	6 (85.7%)	12 (60.0%)
No	11(31.4%)	2 (25.0%)	1 (14.3%)	8 (40.0%)
Roof cover				
Dry straw	21 (60.0%)	8 (100.0%)	7 (100.0%)	6 (30.0%)
Zinc or asbestos	13 (37.1%)	0 (0.0%)	0 (0.0%)	13 (65.0%)
Clay	1 (2.9%)	0 (0.0%)	0 (0.0%)	1 (5.0%)
Wallcovering				
Wood or brick	23 (65.7%)	3 (37.5%)	1 (14.3%)	19 (95.0%)
Dry straw or canvas	12 (34.3%)	5 (62.5%)	6 (85.7%)	1 (5.0%)
Floor				
Dirt floor	28 (80.0%)	8 (100.0%)	7 (100.0%)	13 (65.0%)
Cement or ceramic	6 (17.1%)	0 (0.0%)	0 (0.0%)	6 (30.0%)
Wood	1 (2.9%)	0 (0.0%)	0 (0.0%)	1 (5.0%)
Disposal of human waste				
Collective cesspool	25 (71.4%)	6 (75.0%)	7 (100.0%)	12 (60.0%)
Forest	4 (11.4%)	1 (12.5%)	0 (0.0%)	3 (15.0%)
Individual cesspool	2 (5.7%)	0 (0.0%)	0 (0.0%)	2 (10.0%)
Bathroom outside	2 (5.7%)	0 (0.0%)	0 (0.0%)	2 (10.0%)
River/stream	2 (5.7%)	1 (12.5%)	0 (0.0%)	1 (5.0%)
Water source				
River/stream	32 (91.4%)	8 (100.0%)	7 (100.0%)	17 (85.0%)
Artesian well	3 (8.6%)	0 (0.0%)	0 (0.0%)	3 (15.0%)
Water treatment				
Yes	11 (31.4%)	1 (12.5%)	4 (57.1%)	6 (30.0%)
No	24 (68.6%)	7 (87.5%)	3 (42.9%)	14 (70.0%)

**Table 2:** Sociodemographic and clinical characteristics of the study population, according to villages of residence, *Sawré Muybu* Indigenous Land, Pará, Amazon, Brazil, 2019.

Characteristics	Overall *n* = 200	Village			*p*-Value ^1^
		*Poxo Muybu* *n* = 66	*Sawre Aboy* *n* = 40	*Sawre Muybu* *n* = 94	
Age mean (in years) *Minimum–Maximum*	18 (0–73)	16 (0–59)	17 (0–72)	18 (0–73)	0.820
Sex					0.570
Female	109 (55.0%)	36 (55.0%)	19 (48.0%)	54 (57.0%)	
Male	91 (45.0%)	30 (45.0%)	21 (52.0%)	40 (43.0%)	
Age group					0.100
Under 12 years	88 (44.0%)	31 (47.0%)	16 (40.0%)	41 (44.0%)	
13–19 years	38 (19.0%)	15 (23.0%)	12 (30.0%)	11 (12.0%)	
20–29 years	39 (20.0%)	8 (12.0%)	5 (12.0%)	26 (28.0%)	
30–44 years	21 (10.0%)	8 (12.0%)	5 (12.0%)	8 (8.5%)	
45 years and over	14 (7.0%)	4 (6.1%)	2 (5.0%)	8 (8.5%)	
Schooling					0.390
Illiterate	4 (2.0%)	1 (1.5%)	1 (2.5%)	2 (2.1%)	
1–9 years	142 (71.0%)	42 (64.0%)	31 (78.0%)	69 (73.0%)	
≥10 years	26 (13.0%)	14 (21.0%)	3 (7.5%)	9 (9.6%)	
Not applicable	28 (14.0%)	9 (14.0%)	5 (12.0%)	14 (15.0%)	
Marital status					0.049
Single	30 (15.0%)	15 (23.0%)	9 (22.0%)	6 (6.4%)	
Married	78 (39.0%)	23 (35.0%)	16 (40.0%)	39 (41.0%)	
Widow(er)	6 (3.0%)	1 (1.5%)	1 (2.5%)	4 (4.3%)	
Not applicable	86 (43.0%)	27 (41.0%)	14 (35.0%)	45 (48.0%)	
Anemia					0.054
Yes	66 (33.0%)	22 (33.0%)	19 (48.0%)	25 (27.0%)	
No	129 (64.0%)	42 (64.0%)	19 (48.0%)	68 (72.0%)	
Missing	5 (2.5%)	2 (3.0%)	2 (5.0%)	1 (1.0%)	
Body Mass Index ^2^					0.827
<18.5	4 (3.5%)	2 (5.4%)	1 (4.2%)	1 (1.9%)	
18.5–24.9	73 (63.5%)	22 (59.5%)	17 (70.8%)	34 (63.0%)	
25.0–29.9	34 (29.6%)	11 (29.7%)	5 (20.8%)	18 (33.3%)	
≥30.0	4 (3.5%)	2 (5.4%)	1 (4.2%)	1 (1.9%)	

^1^ Pearson’s Chi-squared test; Fisher’s exact test; ^2^ Only to indigenous ≥12 years old.

**Table 3:** Genotypic distribution of polymorphisms in villages, *Sawré Muybu* Indigenous Land, Pará, Amazon, Brazil, 2019.

Gene/SNP	Village	*n* *	Genotypic Distribution *n* (%)			*p*-Value **
TNF-α (Chromossome 6)						
rs1799964			TT	TC	CC	
	*Poxo Muybu*	61	56 (91.8)	4 (6.6)	1 (1.6)	0.008
	*Sawre Aboy*	40	25 (62.5)	13 (32.5)	2 (5.0)	
	*Sawre Muybu*	97	70 (72.1)	25 (25.8)	2 (2.1)	
rs1799724			CC	CT	TT	
	*Poxo Muybu*	62	31 (50.0)	27 (43.5)	4 (6.5)	0.34
	*Sawre Aboy*	40	15 (37.5)	24 (60.0)	1 (2.5)	
	*Sawre Muybu*	97	50 (51.5)	40 (41.3)	7 (7.2)	
rs1800629			GG	GA	AA	
	*Poxo Muybu*	56	56 (100)	0	0	0.050
	*Sawre Aboy*	40	39 (97.5)	1 (2.5)	0	
	*Sawre Muybu*	97	89 (91.8)	8 (8.2)	0	
IL6 (Chromosome 7)						
rs1800795			GG	GC	CC	
	*Poxo Muybu*	62	62 (100)	0	0	0.59
	*Sawre Aboy*	40	40 (100)	0	0	
	*Sawre Muybu*	97	96 (99.0)	1 (1.0)	0	
ALAD (Chromosome 9)						
rs1800435			CC	CG	GG	
	*Poxo Muybu*	56	56 (100)	0	0	0.36
	*Sawre Aboy*	40	40 (100)	0	0	
	*Sawre Muybu*	96	94 (97.9)	2 (2.1)	0	
GSTP1 (Chromosome 11)						
rs1695			AA	AG	GG	
	*Poxo Muybu*	62	37 (59.7)	24 (38.7)	1 (1.6)	<0.0001
	*Sawre Aboy*	40	30 (75.0)	10 (25.0)	0	
	*Sawre Muybu*	97	13 (13.4)	61 (62.9)	23 (23.7)	
VDR (Chromosome 12)						
rs2228570			CC	CT	TT	
	*Poxo Muybu*	62	27 (43.5)	29 (46.8)	6 (9.7)	0.01
	*Sawre Aboy*	40	9 (22.5)	22 (55)	9 (22.5)	
	*Sawre Muybu*	97	49 (50.5)	41 (42.3)	7 (7.2)	
MMP2 (Chromosome 16)						
rs2285053			CC	CT	TT	
	*Poxo Muybu*	62	58 (93.5)	4 (6.5)	0	<0.0001
	*Sawre Aboy*	40	34 (85.0)	6 (15.0)	0	
	*Sawre Muybu*	96	58 (60.4)	37 (38.5)	1 (1.1)	

* *n* is the number of examined samples of the participants for each polymorphism. Differences in sample sizes are due to available data from PCR amplification for each polymorphism. ** *p*-value from Chi-square test (Pearson *p*-value) or Fisher’s exact test.

**Table 4:** Hair Mercury levels (mean, standard deviation, median, minimum, maximum, and prevalence % of ≥6 µg/g), according to the villages of residence (*Sawré Muybu, Poxo Muybu, Sawré Aboy*), by age group and sex, *Sawré Muybu* Indigenous Land, Pará, Amazon, Brazil, 2019.

	Hair Mercury Levels						
Villages	*n*	Mean	Standard Deviation	Median	Minimum	Maximum	≥6.0 µg/g
*Sawré Muybu*							
Children < 12 years	38	5.9	4.7	4.3	1.6	22.1	28.9
Adults ≥ 12 years							
Male	24	7.3	3.2	6.9	2.6	16.0	66.7
Female	29	6.3	3.5	4.7	2.0	14.1	41.4
Total	91	6.4	4.0	5.2	1.6	22.1	42.9
*Poxo Muybu*							
Children < 12 years	28	5.9	2.6	5.8	1.4	11.8	46.4
Adults ≥ 12 years							
Male	18	7.1	2.3	7.3	2.8	11.9	61.1
Female	20	7.6	2.2	7.3	4.2	12.9	80.0
Total	66	6.8	2.5	6.6	1.4	12.9	60.6
*Sawré Aboy*							
Children < 12 years	15	11.0	5.7	10.1	2.6	23.9	80.0
Adults ≥ 12 years							
Male	14	13.6	5.4	14.2	4.8	22.8	92.9
Female	11	12.1	4.1	11.9	5.0	20.2	90.9
Total	40	12.2	5.3	11.5	2.6	23.9	87.5
All Villages							
Children < 12 years	81	6.9	4.8	5.5	1.4	23.9	44.4
Adults ≥ 12 years							
Male	56	8.8	4.6	7.5	2.6	22.8	71.4
Female	60	7.8	3.8	7.3	2.0	20.2	63.3
Total	197	7.7	4.5	6.6	1.4	23.9	57.9

**Table 5:** Sociodemographic and clinical characteristics of the participants ≥ 12 years old, according to hair mercury exposure (≥6.0 µg/g x < 6.0 µg/g), *Sawré Muybu* Indigenous Land, Pará, Amazon, Brazil, 2019.

	Hair Mercury Levels						
	<6.0 µg/g		≥6.0 µg/g		Total		*p*-Value *
Sociodemographic Characteristics							
Sex	n	% ^#^	n	% ^#^	n	% ^†^	
Female	22	36.7	38	63.3	60	51.7	0.353
Male	16	28.6	40	71.4	56	48.3	
Total	38	32.8	78	67.2	116		
Age range (years)							
12 to 19	13	29.5	31	70.5	44	37.9	0.843
20 to 29	13	34.2	25	65.8	38	32.8	
30 and +	12	35.3	22	64.7	34	29.3	
Total	38		78		116		
Marital Status							
Married	25	33.3	50	66.7	75	64.7	0.980
Single	11	31.4	24	68.6	35	30.2	
Widow(er)	2	33.3	4	66.7	6	5.2	
Total	38		78		116		
Income							
Yes	18	45.0	22	55.0	40	34.5	0.042
No	20	26.3	56	73.7	76	65.5	
Total	38		78		116		
Schooling (years)							
≥10	11	45.8	13	54.2	24	20.7	0.170
5 to 9	23	31.5	50	68.5	73	62.9	
1 to 4	2	13.3	13	86.7	15	12.9	
Iliterate	2	50.0	2	50.0	4	3.4	
Total	38		78		116		
Villages							
*Sawré Muybu*	25	47.2	28	52.8	53	45.7	0.002
*Poxo Muybu*	11	28.9	27	71.1	38	32.8	
*Sawré Aboy*	2	8.0	23	92.0	25	21.6	
Total	38		78		116		
Clinical characteristics							
BMI (kg/m^2^)	n	%	n	%	n	%	
18.5–24.9	24	33.3	48	66.7	72	63.7	0.972
<18.5	1	25.0	3	75.0	4	3.5	
25.0–29.9	11	33.3	22	66.7	33	29.2	
≥30.0	1	25.0	3	25.0	4	3.5	
Total	37		76		113		
Blood Pressure							
Normal	37	36.3	65	63.7	102	88.7	0.039
Hypertension	1	7.7	12	92.3	13	11.3	
Total	38		77		115		
Anemia							
No	32	34.8	60	65.2	92	80.0	0.428
Yes	6	26.1	17	73.9	23	20.0	
Total	38		77		115		
Glucose							
<100.0 mg/dL	2	50.0	2	50.0	4	11.1	0.293
≥100.0 mg/dL	8	25.0	24	75.0	32	88.9	
Total	10		26		36		
Previous Hospitalization							
No	22	32.4	46	67.6	68	58.6	0.912
Yes	16	33.3	32	66.7	48	41.4	
Total	38		78		116		

* Pearson’s Chi-Square; ^#^ % in row; ^†^ % in column.

**Table 6:** Hg levels in women of childbearing age, according to the number of children, pregnancy, and breastfeeding status, *Sawré Muybu* Indigenous Land, Pará state, Brazilian.

	Hair Mercury Levels						
Women Features	<6.0 µg/g		≥6.0 µg/g		Total		*p*-Value *
Number of children	**n**	% ^#^	**n**	% ^#^	**n**	% ^†^	
1 to 2	4	28.6	10	71.4	14	26.4	0.531
3 to 6	8	50.0	8	50.0	16	30.2	
7 or +	1	20.0	4	80.0	5	9.4	
No children	7	38.9	11	61.1	18	34.0	
Total	20	37.7	33	62.3	53		
Pregnant							
No	20	41.7	28	58.3	48	90.6	0.067
Yes	0	0.0	5	100.0	5	9.4	
Total	20		33		53		
Breastfeeding							
No	13	36.1	23	63.9	36	67.9	0.723
Yes	7	41.2	10	58.8	17	32.1	
Total	20		33		53		

* Pearson’s Chi-Square; ^#^ % in row; ^†^ % in column. Obs: Hg mean level in pregnant = 8.86 µg/g; Hg mean level in non-pregnant = 7.59 µg/g (F-test: 1.94; *p*-value: 0.169).

**Table 7:** Clinical characteristics of Munduruku adults ≥ 12 years old, including height (cm), weight (kg), SBP and DBP (mmHg), glucose level (mg/dL), and hemoglobin level (g/dL), according to mercury exposure (<6.0 µg/g x ≥ 6.0 µg/g), *Sawré Muybu* Indigenous Land, Pará, Amazon, Brazil, 2019.

Clinical Characteristics	Hg Detected	Participants	Mean	SD ^#^	SE ^†^	F-Test	*p*-Value
Height (cm)	<6.0 µg/g	37	150.6	6.7916	1.1165	0.065	0.799
	≥6.0 µg/g	76	151.2	7.2156	0.8277		
Weight (kg)	<6.0 µg/g	37	53.7	9.5022	1.5621	1.189	0.278
	≥6.0 µg/g	77	52.8	9.7792	1.1144		
BMI (kg/m^2^)	<6.0 µg/g	37	23.6	3.38	0.5557	1.936	0.167
	≥6.0 µg/g	76	23.1	3.6339	0.4168		
SBP * (mmHg)	<6.0 µg/g	38	110.5	9.42	1.528	4.157	0.044
	≥6.0 µg/g	77	113.2	15.557	1.773		
DBP ** (mmHg)	<6.0 µg/g	38	67.8	7.577	1.229	4.363	0.039
	≥6.0 µg/g	77	69.3	11.225	1.279		
Glucose level (mg/dL)	<6.0 µg/g	10	115.2	16.858	5.331	0.075	0.786
	≥6.0 µg/g	26	115.4	19.438	3.812		
Hemoglobin level (g/dL)	<6.0 µg/g	38	13.8	1.2437	0.2018	0.175	0.677
	≥6.0 µg/g	77	13.8	1.3299	0.1516		

* Systolic Blood Pressure; ** Diastolic Blood Pressure; ^#^ Standard Deviation; ^†^ Standard Error.

**Table 8:** Logistic regression model based on prevalence ratios (PR) crude and adjusted (confidence interval 95%), according to the mercury exposure (≥6.0 µg/g), to indigenous ≥12 years old, and to women of childbearing age, *Sawré Muybu* Indigenous Land, Pará, Amazon, Brazil, 2019.

**Indigenous ≥ 12 Years-Old Both Sex**						
**Characteristics**	**Crude PR**	**95%CI**	***p*-Value**	**Adjusted PR**	**95%CI**	***p*-Value**
Villages						
*Sawré Muybu*	1			1		
*Poxo Muybu*	1.3	(0.9–1.9)	0.074	1.3	(0.9–1.8)	0.098
*Sawré Aboy*	1.7	(1.3–2.3)	0.001	1.8	(1.3–2.3)	0.001
Income						
Yes	1			1		
No	1.3	(0.9–1.8)	0.065	1.3	(1.0–1.8)	0.031
Schooling (years)						
≥10	1					
5 to 9	1.3	(0.9–1.9)	0.250	--		
1 to 4	1.6	(1.0–2.4)	0.028	--		
Illiterate	0.9	(0.3–2.6)	0.881	--		
Blood Pressure						
Normal	1					
Hypertension	1.4	(1.2–1.8)	0.001	1.6	(1.3–2.1)	0.001
**Women of Childbearing Age**						
**Characteristics**	**Crude PR**	**95%CI**	***p*-Value**	**Adjusted PR**	**95%CI**	**p-Value**
Villages						
*Sawré Muybu*	1			1		
*Poxo Muybu*	2.1	(1.2–3.7)	0.010	1.9	(1.0–3.4)	0.044
*Sawré Aboy*	2.4	(1.4–4.2)	0.002	2.5	(1.4–4.4)	0.001
Income						
Yes	1			--		
No	1.3	(0.8–2.3)	0.273	--		
Blood Pressure						
Normal	1			1		
Hypertension	1.7	(1.3–2.1)	0.001	1.9	(1.2–3.3)	0.012
Pregnant status						
No	1			1		
Yes	1.7	(1.4–2.2)	0.001	1.5	(1.0–2.1)	0.029

## Data Availability

Data sharing not applicable.
